# Psoriatic arthritis incidence and prevalence trajectory in Poland—public-payer, national-level, long-term data

**DOI:** 10.1007/s00296-025-05966-5

**Published:** 2025-09-02

**Authors:** Magdalena Krajewska-Włodarczyk, Mateusz Szeląg, Bogdan Batko, Marcin Stajszczyk, Michał Orleański, Krzysztof Podwójcic, Jakub Sowiński, Maria Świderek, Marek Brzosko, Agata Śmiglewska, Brygida Kwiatkowska, Zbigniew Żuber

**Affiliations:** 1 Clinic of Rheumatology, School of Medicine, Collegium Medicum, University of Warmia and Mazury, Olsztyn, Poland; 2 Department of Analysis and Strategy, Ministry of Health, Warsaw, Poland; 3 Department of Rheumatology and Immunology, Faculty of Medicine and Health Sciences, Andrzej Frycz Modrzewski University, Krakow, Poland; 4 Department of Rheumatology and Autoimmune Diseases, Silesian Center for Rheumatology, Ustroń, Poland; 5 Institute of Labour and Social Studies, Warsaw, Poland; 6 Department of Rheumatology, Internal Diseases, Geriatrics and Clinical Immunology, Faculty of Medicine and Dentistry, Pomeranian Medical University, Szczecin, Poland; 7 Clinic of Early Arthritis, National Institute of Geriatrics, Rheumatology and Rehabilitation, Warsaw, Poland; 8 Department of Pediatrics, Faculty of Medicine and Health Sciences, Andrzej Frycz Modrzewski University, Krakow, Poland

**Keywords:** Psoriatic arthritis, Epidemiology, Incidence, Prevalence, Public health, Administrative data

## Abstract

To estimate the actual incidence and prevalence of psoriatic arthritis (PsA) within a 9-year timeframe in Poland. Patients were defined as having PsA if they had at least two visits more than 90 days apart with ICD-10 codes M07.0, M07.1, M07.2, M07.3, or L40.5 and filled at least one reimbursed prescription for peripheral or axial PsA-specific treatments during this period (including methotrexate, sulfasalazine, ciclosporin, leflunomide, biologics, targeted synthetic drugs, or non-steroidal anti-inflammatory drugs). Data was obtained from the nationwide public payer database, considering gender, age, and region of residence. We observed an incidence rate of 1.1 per 100,000 inhabitants in 2021, compared to 13.2 in 2013. Regarding the age of the first diagnosis, the peak incidence rate decreased, with a more pronounced decline in men. The prevalence of PsA rose from 72.5 individuals per 100,000 in 2013 to 95.5 in 2021, representing approximately 0.1% of the total population in Poland, with a significant predominance of women among patients over 55 years of age. The decline in PsA incidence may be influenced by a strict case definition and improved access to treatment. Higher prevalence in older women suggests potential gender-related differences. The lower peak incidence and younger diagnosis age in men raise questions about whether lower PsA prevalence in older males is linked to higher mortality due to longer disease duration and comorbidities. Further research is needed to clarify these findings.

## Introduction

Psoriatic arthritis (PsA) is an inflammatory joint disease with a variable clinical presentation, most often, but not always, associated with the presence of skin or nail psoriasis. Due to the heterogeneous nature of symptoms, diagnosing PsA can be challenging, which may lead to under- or overestimation of incidence and prevalence data [1]. The prevalence of PsA in published studies ranges from 0.05% [2] to 0.25% [3] in the general population, affecting between 6% and 41% of patients with psoriasis [4]. The highest prevalence of PsA (0.26%) was reported in studies based on self-reports [5]. The prevalence of PsA worldwide is estimated to be approximately 112/100,000 adults, although there is currently considerable variation due to the methodology of the studies conducted [6] and geographical location [5], and ranges in studies based solely on ICD-10 codes from 3.3/100,000 [7] to 461.5/100,000 [8]. In studies using administrative data, the prevalence of PsA averages 175/100,000 population in Europe and 147/100,000 population in North America, while even greater differences in PsA prevalence are seen in population-based studies (207/100,000 and 64/100,000 population in Europe and North America, respectively) [6].

Planning strategies for the medical care of patients with PsA requires a comprehensive assessment of the severity of the problem. In practice, such an assessment is challenging due to the diversity of symptoms, the lack of diagnostic tests and the potential coexistence of other joint diseases in people with psoriasis. Well-designed population-based studies can provide data that are representative of the population as a whole, in addition to collecting information on demographics, lifestyle and environmental factors. Such studies can allow analysis of cause-and-effect relationships, but they can be time-consuming and costly, which may limit the recruitment of participants. Surveys based on administrative data, e.g. based on an ICD-10 code, may even cover the entire study population, which increases the statistical power of the results obtained and offers the possibility of assessing long-term trends, but it should be borne in mind that the quality of data obtained in this way may be heterogeneous and subject to error [9]. The choice between population-based surveys and surveys based on administrative data depends on the objectives of the study and the specificity of the problem being analysed [10]. Administrative health data, such as records of hospitalizations and prescription medications, are primarily generated for administrative purposes, such as billing and healthcare management, rather than being driven by predefined research objectives. Nevertheless, these datasets can be effectively repurposed to facilitate epidemiological studies [11]. While there are relatively new studies based on single or complex administrative data on PsA epidemiology in some Western European countries [12–15], comprehensive, nationwide studies based on complete case definitions are currently lacking. Previous estimates in Poland have been limited by reliance on single-source data [16].

This study addresses this critical gap by providing a robust, nationwide analysis of PsA incidence and prevalence trends in Poland over a nine-year period (2013–2021). By utilizing comprehensive administrative data from the National Health Fund (NFH) and employing a strict case definition incorporating both diagnostic codes and treatment data, this study provides a more accurate and reliable assessment of current PsA epidemiology in Poland than previously available. The findings of this study may be of direct benefit to clinicians, providing a contemporary understanding of disease burden and trends, which can inform diagnostic and treatment decisions. Furthermore, health policy makers can leverage these results to optimize resource allocation and improve the quality of care for individuals with PsA in Poland. Specifically, the analysis of incidence trends, age of diagnosis, and gender disparities may inform public health initiatives aimed at early detection.

## Materials and methods

We utilized electronic administrative health claims collected from 2009 to 2022 by the National Health Fund, a single public healthcare payer in Poland. The NHF database comprises individually reported data submitted to the payer by service providers, including detailed service descriptions and demographic variables that describe the patient. The data collected in databases are anonymous, but individual patients can be distinguished and matched by their IDs, which are pseudonymized national identification numbers.

### Psoriatic arthritis definition

Information on reported NHF health services and reimbursed prescriptions was used to define people with psoriatic arthritis.

To exclude initial diagnoses reported as ICD-10 M07.0, M07.1, M07.2, M07.3 or L40.5 but not confirmed by further diagnosis, we developed a working definition of people with psoriatic arthritis. PsA patients were defined as persons who had at least two visits with ICD-10 codes M07.0, M07.1, M07.2, M07.3 or L40.5 in any type of service (outpatient or inpatient) more than 90 days apart and at the same time filled at least one reimbursable prescription for a drug whose active ingredient is methotrexate (ATC L01BA01, ATC L04AX03) or leflunomide (ATC L04AA13) or sulfasalazine (ATC A07EC01) or ciclosporin (ATC L04AD01, ATC S01XA18) or non-steroidal anti-inflammatory drugs (NSAIDs) including diclofenac (ATC M01AB05), piroxicam (ATC M01AC01), lornoxicam (ATC M01AC05), meloxicam (ATC M01AC06), ibuprofen (ATC M01AE01), naproxen (ATC M01AE02), ketoprofen (ATC M01AE03), tiaprofenic acid (ATC M01AE11), dexibuprofen (ATC M01AE14), dexketoprofen (ATC M01AE17), mefenamic acid (ATC M01AG01), celecoxib (ATC M01AH01), rofecoxib (ATC M01AH02), etoricoxib (ATC M01AH05) or biologics including etanercept (ATC L04AB01), infliximab (ATC L04AB02), adalimumab (ATC L04AB04), certolizumab pegol (ATC L04AB05), golimumab (ATC L04AB06), secukinumab (ATC L04AC10), ixekizumab (ATC L04AC13)) or targeted synthetic disease-modifying antirheumatic drug (tsDMRDs) tofacitinib (ATC L04AF01). Due to the lack of reimbursement in Poland in the studied period, the analysis did not include some biologics and tsDMRDs as guselkumab (ATC L04AC16), risankizumab (ATC L04AC18), bimekizumab (ATC L04AC21), ustekinumab (ATC L04AC05) and upadacitinib (ATC L04AF03).

The date of diagnosis was considered the date of either the first treatment prescription or the first visit, whichever occurred earlier. The rationale for this approach is the possibility that a reimbursed prescription may have been issued during a private health care visit, as visits within the private health care system are not reported to the NHF, and completed reimbursed prescriptions, whether issued within the public or private health care system, are visible in the NHF databases. According to current standards for diagnosing PsA, the disease can be confirmed in individuals aged 16 years or older.

### Patients

Databases from 2009 onwards were used to identify patients and determine their date of first diagnosis. However, due to access to reimbursed treatment data only since 2012, we assessed incidence from 2013 onwards to avoid the error of overestimating the incidence of PsA in 2012. Patients were classified as living in the urban or rural region based on the correct National Official Register of the Territorial Division of the Country (TERYT) code reported in the medical history of their first NHF-registered outpatient visit or hospitalization.

### Registered incidence and prevalence

Registered incidence was defined as the number of new patients with a diagnosis of PsA reported through the publicly funded healthcare system in a calendar year. Registered prevalence in a given year was defined as the number of PsA patients alive on 31 December of that year in the NHF database. The Ministry of Digital Affairs’ death register [17] and information on the total population of Poland, provided by the Central Statistical Office [18], were used to determine the prevalence.

### Statistics

All database querying, data processing, computations, and visualisations were conducted using the GNU R programming language within the RStudio environment. Discrete variables were summarized with counts and proportions (N, %).

The prevalence of PsA in Poland was calculated based on the presented NHF data and additional demographic data, including the number of Polish inhabitants, made available by the Central Statistical Office [18].

To calculate standardised incidence and prevalence, we use data from the Central Statistical Office regarding the population size of a specific year. We employed standardised indicators for the entire Polish population to compare incidence and prevalence in Poland with those of other highly developed countries.

## Results

### Incidence of PsA

**Fig. 1:**
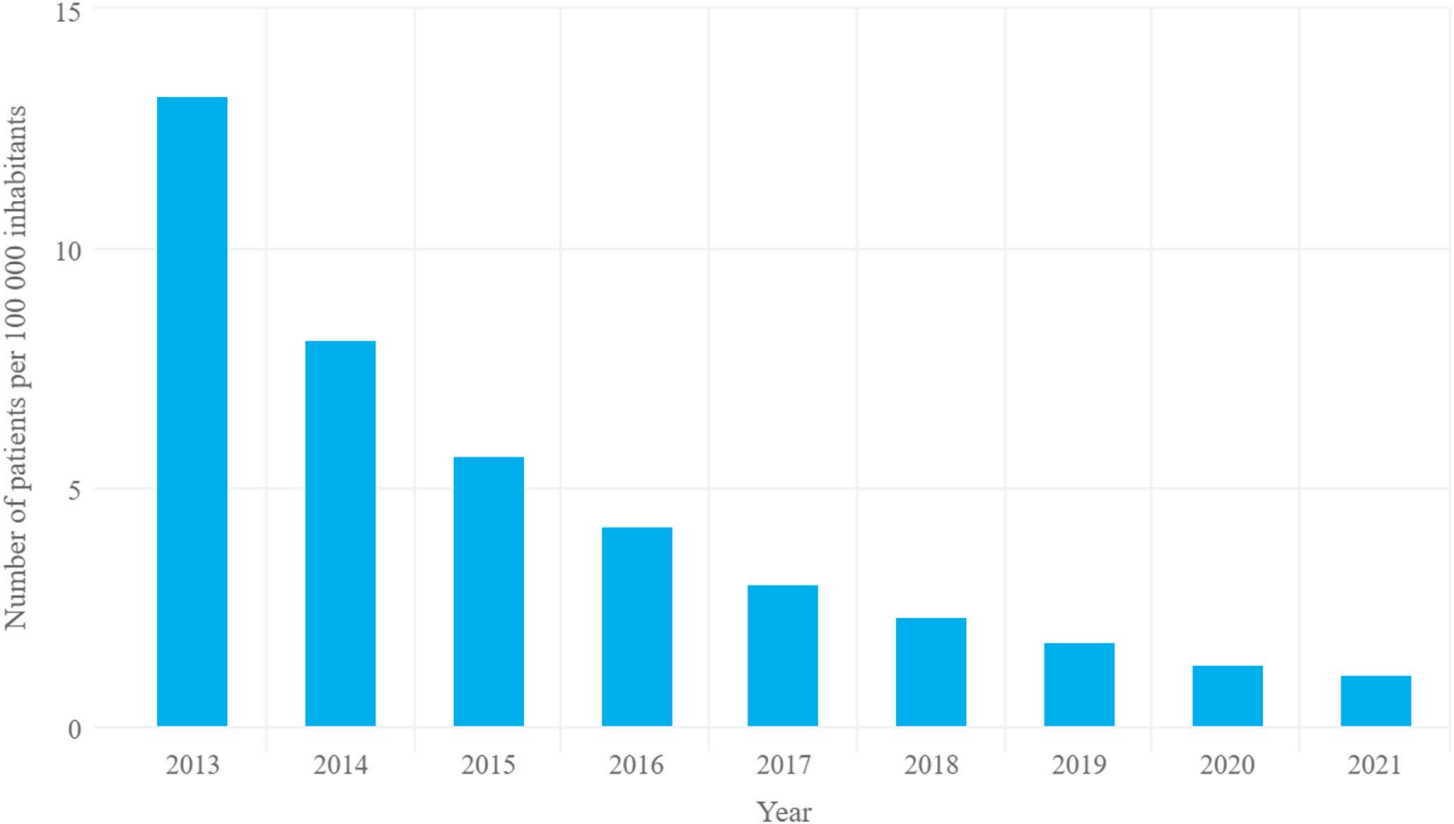
Registered incidence between 2013 and 2021 in the general population per 100,000 population

In 2021, 431 people were diagnosed with PsA in Poland, i.e. 1.1 per 100,000 people. In the period under analysis, the annual number of new diagnoses of PsA decreased. In 2013, the disease was diagnosed in 5098 people, which corresponds to 13.2 people per 100,000 population (Fig. 1).

## Age at first diagnosis

**Fig. 2:**
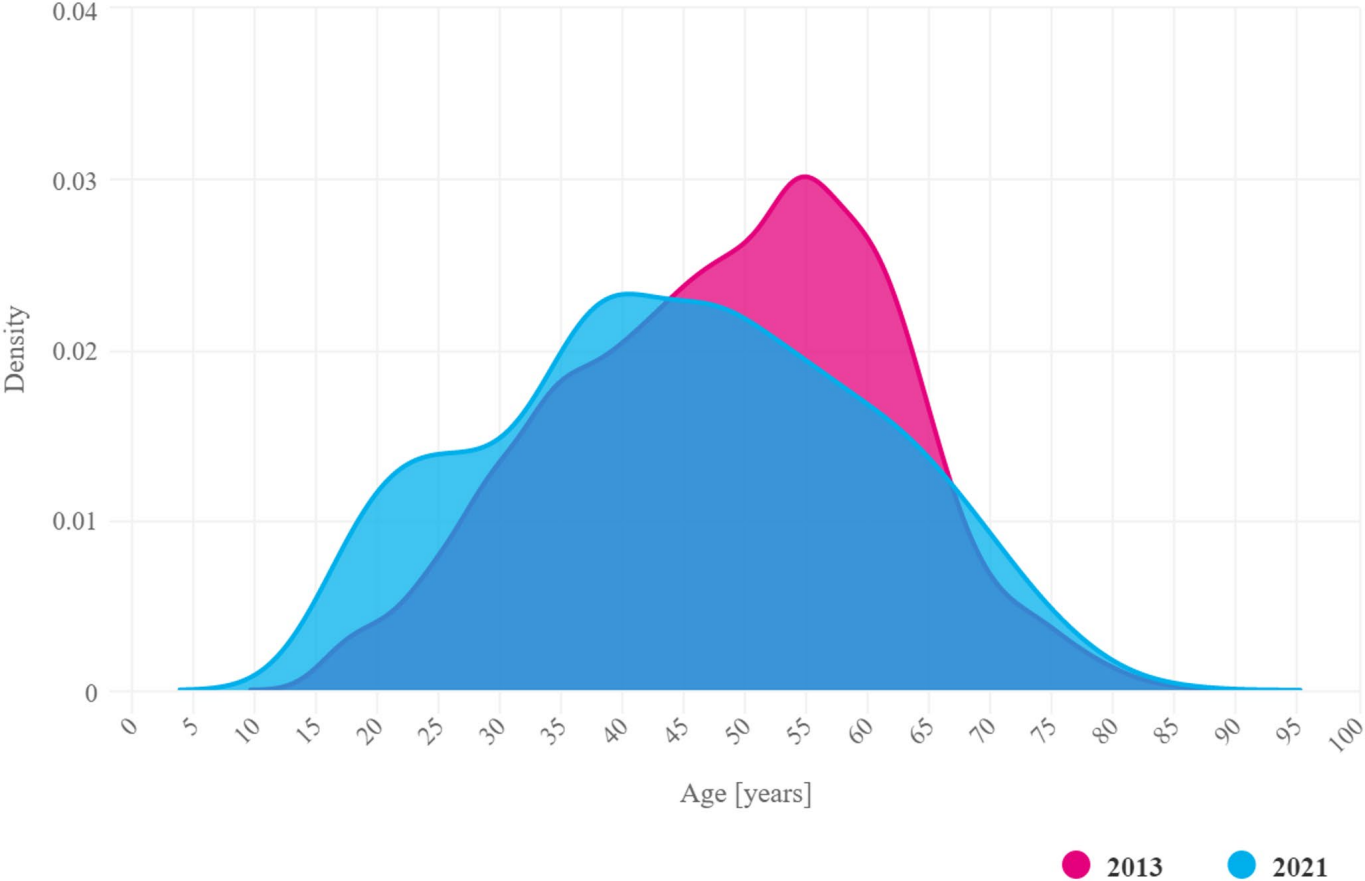
Age distribution of the incidence of PsA in the Polish population in 2013 and 2021

Between 2013 and 2021, the majority of new diagnoses of PsA were made in patients aged 35–55 years, regardless of gender. In 2021, the peak incidence of PsA occurred around the age of 45. (mean 44.8; median 45), earlier than in 2013. (mean 48.5; median 50). (Fig. 2).

**Fig. 3:**
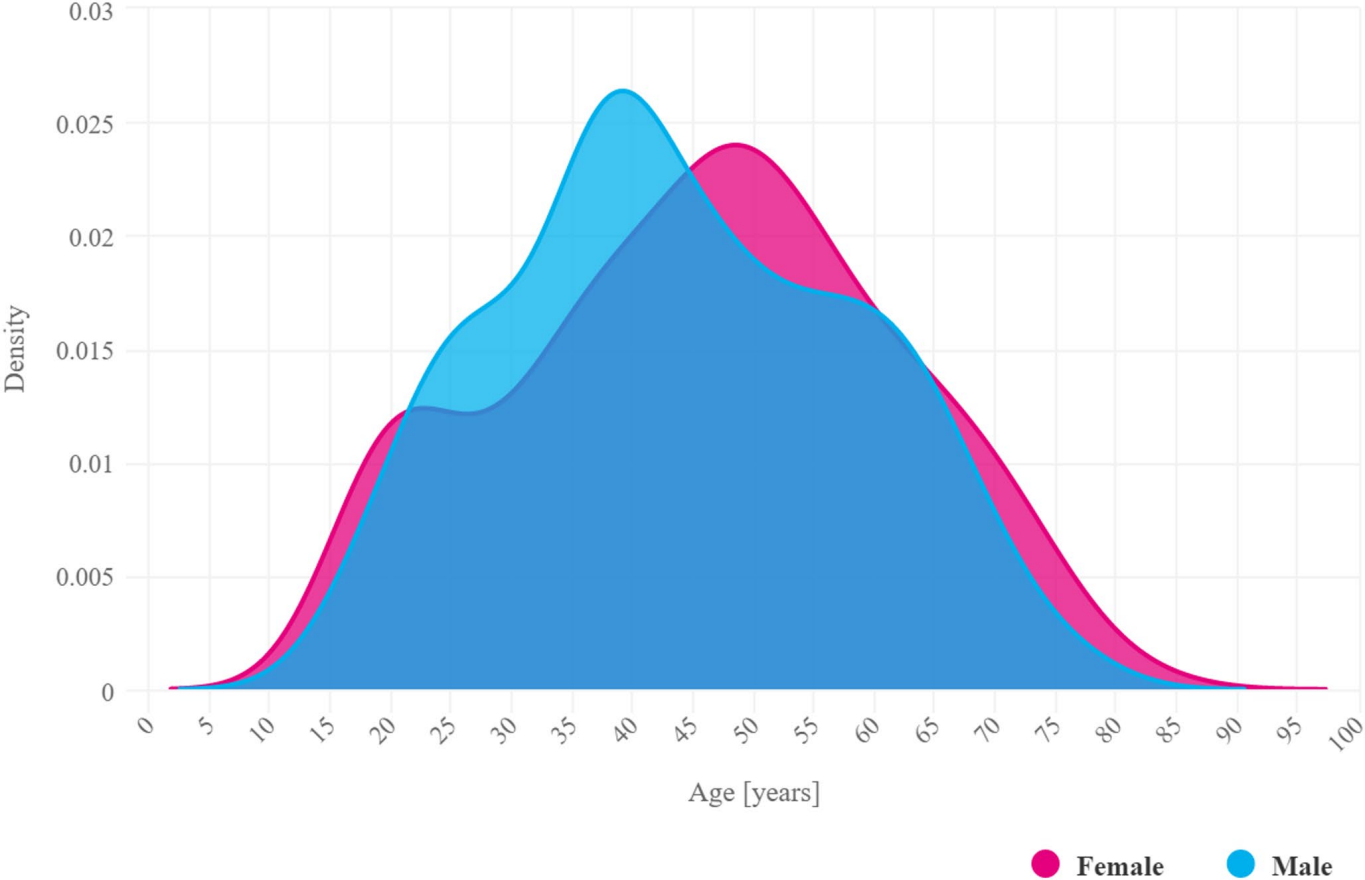
Age distribution of incidence of PsA in 2021 by gender

We also observed a clear trend towards earlier incidence in men. In 2021, the peak incidence was 48 years for women and 39 years for men. (Fig. 3). The median age of first diagnosis in 2021 was 47 years for women and 42 years for men.

Additionally, a slightly earlier age of onset of PsA was observed in rural areas. In 2021, the median age of diagnosis of PsA was 45 years in urban areas and 42 years in rural areas.

## The prevalence of PsA in Poland

**Fig. 4:**
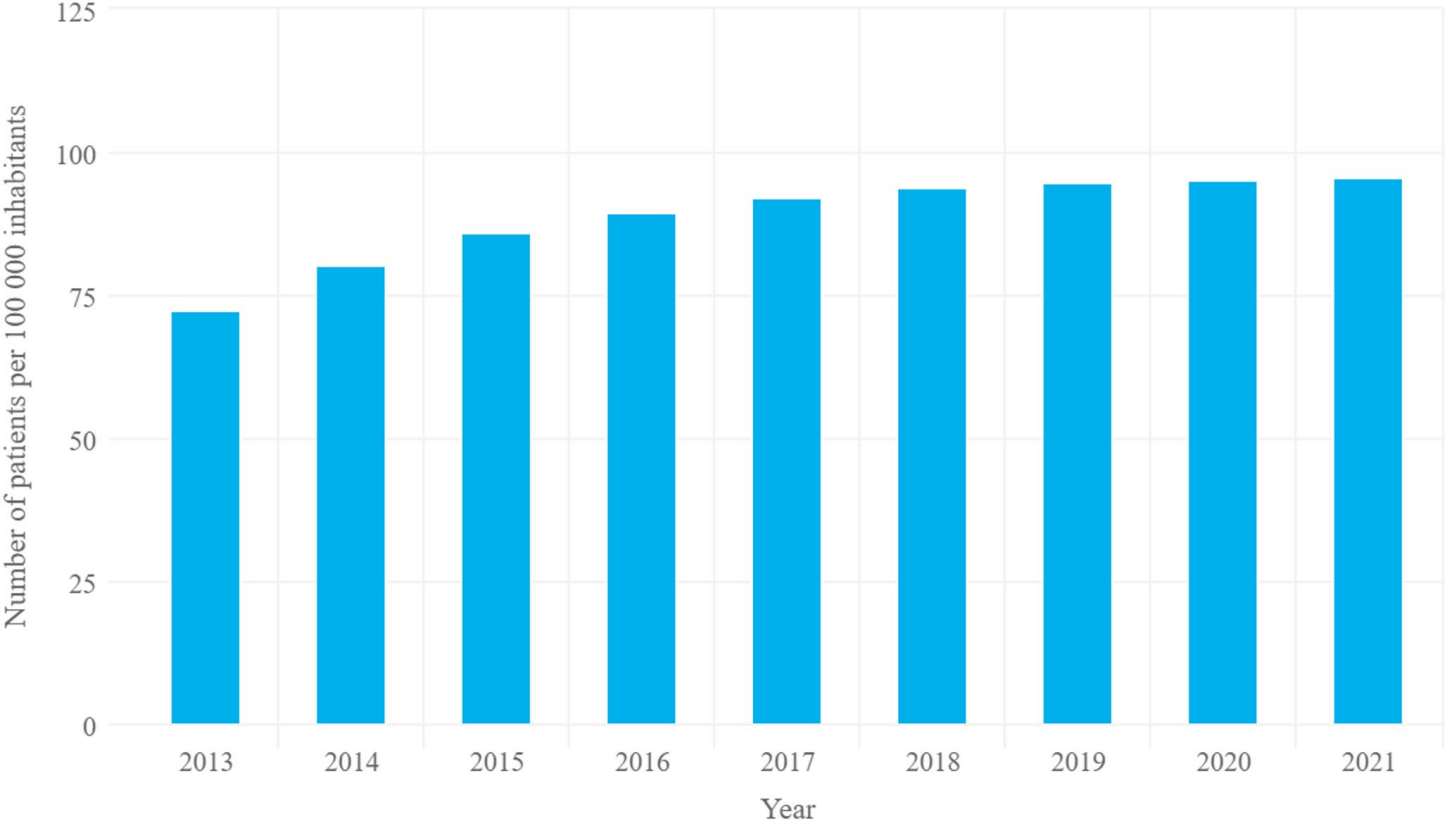
Registered morbidity from 2013 to 2021 in the general population per 100,000 population

The prevalence of PsA in Poland was estimated at 0.098% in 2021, and the total number of living patients with PsA in Poland was 36,357 (31 December 2021), representing 95.5 per 100,000 population. A notable increase in prevalence was observed across the entire Polish population during the assessed period (Fig. 4). The largest proportion of patients with PsA was observed among individuals aged 55–64 years (27% of all patients with PsA), while the smallest proportion was observed among patients younger than 18 years (0.2% of the total number of patients).

**Fig. 5:**
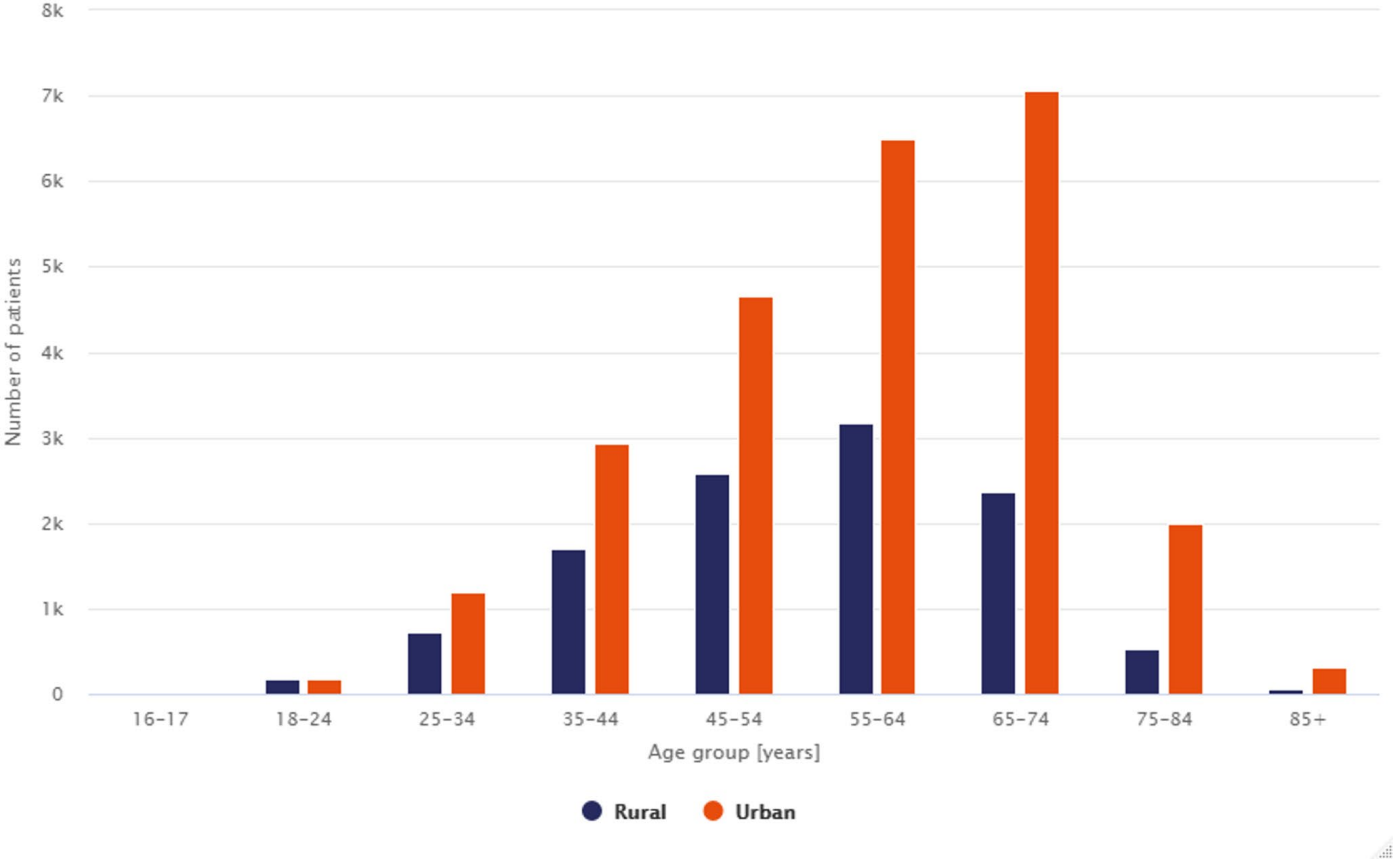
Number of all patients in Poland by age group and area of residence

Patients with PsA in Poland mainly live in urban areas − 69% of the total number of patients. This distribution is similar to that of the Polish population. The mean and median ages of patients living in urban areas (mean: 57.3 years, median: 60 years) are 3.5 and 5 years higher, respectively, than those living in rural areas. Half of all patients with PsA are urban dwellers aged 45–74 years (Fig. 5). The differences in the age structure of patients with PsA living in urban and rural areas reflect differences in the age structure of the Polish population as a whole.

### Prevalence by gender

**Fig. 6:**
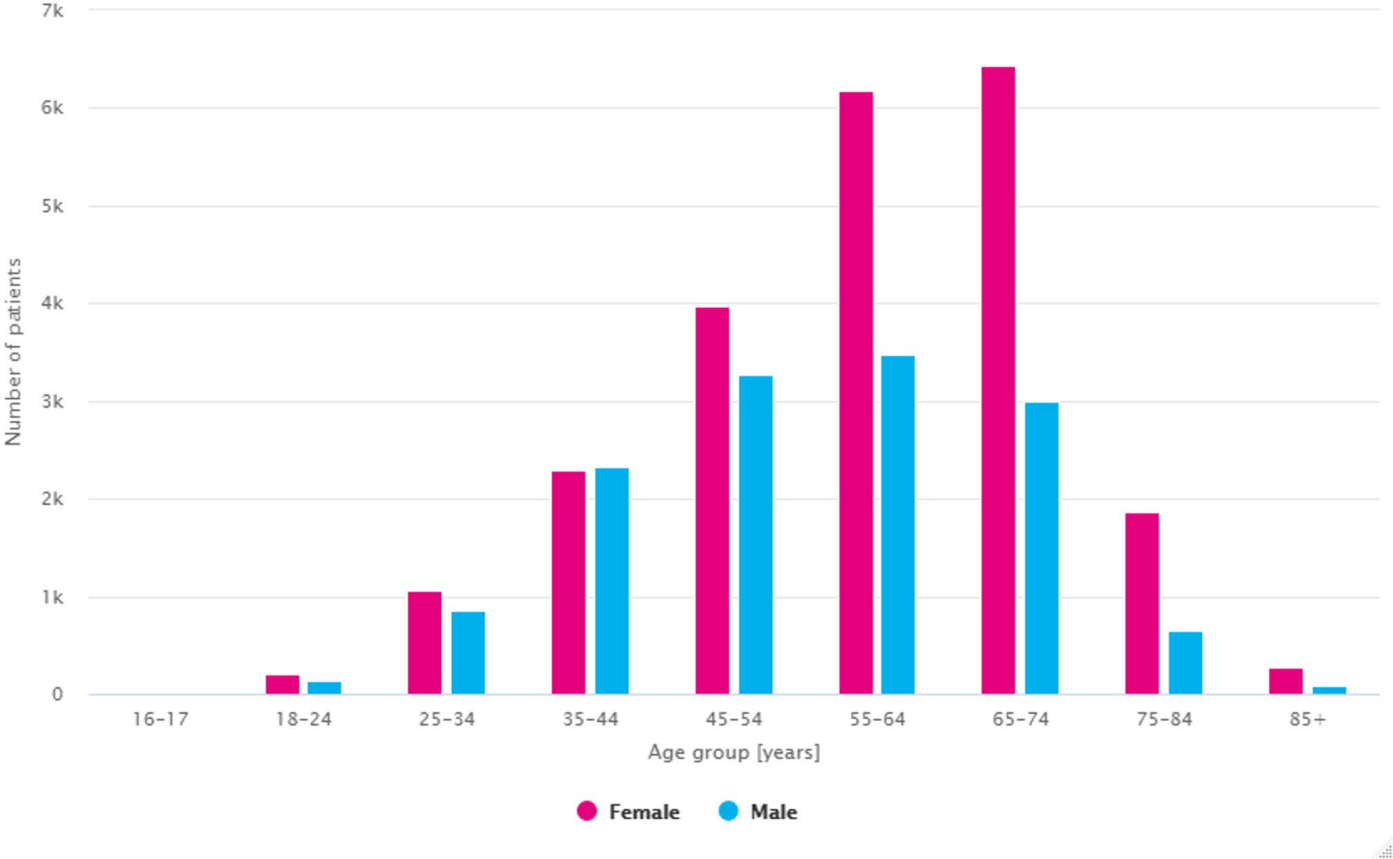
Number of all patients with PsA in Poland in 2021 by age group and gender

The highest prevalence in 2021, for both men and women, was in the sixth decade of life (Fig. 6). In 2021, about 61% of the total number of people diagnosed with PsA were women, with the percentage distribution changing with age. The number of female and male patients was relatively equal between 18 and 44 years of age, with a significant increase in female prevalence after 55 years of age – women accounted for 65% of patients in the 55–64 age group, 70% in the 65–74 age group and about 75% after 75 years of age.

## Discussion

The aim of our study was to assess the incidence and prevalence of PsA in Poland from 2013 to 2021 using administrative data from the National Health Fund in Poland. The study design aimed to exclude misdiagnosis as much as possible, so a strict patient definition was developed, taking into account simultaneously reported ICD-10 codes and PsA-dedicated treatment prescription fulfilment. We recognize that these exclusions could disproportionately affect certain subgroups of PsA patients and might introduce bias into our prevalence and incidence estimates. To mitigate this concern, we considered the balancing of specificity and sensitivity. The decision to use a stringent definition was a deliberate trade-off between specificity and sensitivity. We prioritized specificity to minimize the inclusion of false-positive cases, particularly given the reliance on administrative data and the potential for coding errors.

We reported an incidence rate for PsA in 2021 of 1.1 per 100,000 inhabitants, which is more than ten times lower than in 2013. The most significant decline in newly diagnosed cases occurred between 2013 and 2017. Our analysis revealed gender differences in the age of first diagnosis throughout the study period, with peak incidence occurring nine years earlier for men. There were no significant differences in incidence between rural and urban areas. In contrast to incidence, there was a trend of increasing prevalence over the analysed period, most notably between 2013 and 2018, with numbers stabilizing in subsequent years. Data from the last analyzed year indicated that women make up the majority of the entire PsA patient population, clearly evident across all age groups starting from 55 years old.

The marked decline in incidence is challenging to explain, but several potential factors should be considered. Firstly, it is possible that the higher incidence reported in 2013 reflects a primary overestimation due to the methodology of utilizing administrative data. At the beginning of the study period, the database might have included prevalent cases (patients already diagnosed before 2013) rather than solely representing new diagnoses within that year. The two-visit and one-prescription criteria, while aiming to ensure diagnostic accuracy, might not fully differentiate between new and existing cases at the beginning of the study period. We have attempted to mitigate this issue by including two visits with ICD-10 codes and filling at least one reimbursed prescription in the case definition. This was based on the assumption that patients with ongoing care are more likely to be properly diagnosed and identified in the database. However, we recognize that some individuals with pre-existing diagnoses may have met these criteria in 2013, contributing to an inflated initial incidence rate.

However, we would like to highlight the significance of the relatively stable decrease in incidence rates observed in the later years of the study. After the initial sharp decline, the incidence appears to have stabilized at a trend, suggesting that the true new case incidence may be closer to the values observed in 2019–2021. It is also essential to emphasize that interpreting studies using administrative data necessitates careful consideration. While administrative databases provide valuable insights into population-level trends, they also present limitations related to coding accuracy, changes in diagnostic practices, and the inherent challenges of distinguishing between incident and prevalent cases, particularly at the beginning of the study period. Another possible explanation could be the previous overdiagnosis of psoriatic arthritis, possibly due to an earlier tendency to classify any arthritis or joint pain in patients with psoriasis as PsA. This may have been related to the slow introduction of the Classification of Arthritis in Psoriasis (CASPAR) criteria at that time [19]. A noticeable lifestyle change is also observed, particularly among younger Poles, including a reduction in smoking and alcohol consumption - environmental factors that are often cited [20]. Additionally, the lower prevalence of obesity in the general population among adult men and women in 2019 compared to 2013–2014 [21], a recognized independent risk factor for PsA [22], might also be of interest, although we have no data on whether this applies to a subpopulation with psoriasis. Another important aspect is the potential prevention of PsA development in patients with psoriasis resulting from the treatment used, including biologics [23]. The increasing use of systemic treatment for severe psoriasis, including biological treatments, may also be significant in the Polish population since the number of psoriatic patients treated biologically increased from about 200 in 2013 to over 2000 in 2021 (based on unpublished NHF data). It can also not be ruled out that some patients with psoriasis treated biologically at dermatological centers meet the criteria for diagnosing PsA, but due to the effective treatment of skin and joint lesions under the care of a dermatologist, the diagnosis of PsA was not added. According to the NHF data, these patients are still categorized only as having psoriasis. While the significantly increased availability of biological treatments for psoriasis after 2019 may partially explain the latest incidence data, it does not account for the substantial decrease in the number of new patients between 2013 and 2017 by approximately 4000. Despite attempts to validate potential contributing factors through existing literature and data, the exact causes for the observed decline remain speculative.

Worldwide data on the incidence of PsA in the general population are limited and largely outdated. Additionally, there are significant variations in published studies depending on the methodology and geographical location. In Europe, the highest incidence (41.3 per 100,000 population) was reported in Norway in 2015, based on a survey conducted between 2000 and 2008 [24]. This contrasts sharply with the results of another Norwegian population-based survey conducted between 1978 and 1996, which reported 6.9 per 100,000 population [25]. A high incidence (23 per 100,000 population) was also observed in Scandinavia in a Finnish survey conducted in 2000 [26]. PsA was diagnosed much less frequently in Central and Southern Europe. In the population of two regions in the Czech Republic, a survey based on administrative data conducted in 2002–2003 reported 3.6 new cases per 100,000 inhabitants [3]. A similar incidence was documented in a comparable study conducted in Greece between 1982 and 2001 (3.02 per 100,000 population) [27] and in a Turkish retrospective study between 2003 and 2012 (2.8 per 100,000 population) [28]. In a recent European study utilizing administrative data from the French National Health Insurance Database and the French National Hospital Discharge Database, the incidence, determined by disease code assigned in hospital or outpatient care (one hospital or outpatient visit was sufficient), was 8.4 per 100,000 population from 2015 to 2018 [12]. In their study, Scott et al. qualified cases of patients with a single diagnosis code assigned by a primary care physician. The incidence in this study increased from 12.2 per 100,000 in 2012 to 17. 2 per 100,000 in 2019, before sharply falling to 5.6 per 100,000 in 2020 [13]. Non-European reports on the magnitude of PsA incidence also vary. The lowest incidence was documented in a Japanese survey-based report (0.1 per 100,000) [29]. A retrospective North American study based on data from 1970 to 1999 in Olmsted County, Minnesota, USA, estimated the incidence at 7.2 per 100,000 population [30], as did a South American paper estimating the incidence of PsA from 2000 to 2005 at 8.3 per 100,000 [31] and an Israeli study using data from the Clalit Health Services (CHS) database, where the incidence averaged 10. 9 per 100,000 from 2006 to 2015 [32]. Direct comparisons of PsA incidence trends observed in our work and other studies [13] are challenging due to significant methodological differences, including variations in case definitions and reporting methods. Unfortunately, there is a paucity of comparable, long-term data on PsA incidence across different countries. Many studies focus on prevalence rather than incidence. Further research is needed to establish whether declining PsA incidence trends have been observed elsewhere. The lack of standardized methodology makes it difficult to determine whether the sharp decline observed in Poland is truly unique or simply a reflection of these methodological differences.

We observed the peak incidence of PsA in 2021 at 45 years of age, and most new diagnoses were made between 35 and 55 years of age, slightly earlier than in the studies by Alamanos et al. [27] and Scott et al. [13], where the peak incidence was between 45 and 64 years of age.

More studies have been published on the prevalence of PsA. In our study, the prevalence has been increasing since 2013 and was 95.5 per 100,000 population in 2021, representing about 0.1% of the general population, of which more than 60% were women. The highest prevalence in 2021 was in women’s and men’s sixth decade of life. A previous study of the Polish population, using only ICD-10 codes without additional conditions, estimated a prevalence of 73.1 per 100,000 in 2018, representing 0.073% of the population [16], which is consistent with our 2013 findings. In a recent meta-analysis using administrative data studies, the estimated mean prevalence of PsA worldwide was 109/100,000 [6]. Similar to population-based studies, significant differences in prevalence are observed in studies based on administrative data. In population-based studies, prevalence ranges from 13/100,000 in Kuwait, in a study using questionnaires with subsequent physician confirmation [33], to 580/100,000 in Spain, in a study using telephone interview data confirmed by discussion with a physician [34]. Morbidity in European studies using administrative data ranged from 56.6/100,000 of the Greek population [27] to 461.5/100,000 in a Norwegian study based on a patient definition requiring at least two visits with the corresponding ICD-10 code [8]. In three other European studies using ICD-10 codes without an elaborate patient definition based on a single disease code reported to the database, the prevalence was 0.32% in the Danish population [14], 0.3% in the German population [15] and 0.28% in the United Kingdom [13]. In our study, despite the decrease in incidence, we observed a significant 30% increase in prevalence from 72.5/100,000 in 2013 to 95.5/100,000 in 2021. One possible explanation is improving patient survival, which might be associated with better treatment of the underlying disease and comorbidities. Other studies have reported increasing trends, even a doubling of the number of patients over the observed periods [13, 32]. In our study, we observed the growing prevalence in the general population and the growing percentage of women with PsA in older age groups, which may indicate a better survival rate in women and a lower survival rate in men in the observed period. The younger age at diagnosis of PsA, and therefore longer disease duration in men may lead to a higher rate of comorbidities after age 55 [35], which might impact survival, but this would require confirmation in further studies. Such relevant differences in prevalence do not occur in people under 55 years of age, and the absolute number of PsA men decreases after the age of 65, while the number of women is still stable.

In our study, women were more frequently affected by PsA, accounting for 61% of all patients, which aligns with findings from European studies, including the population-based study by Dönmez et al. [28] and studies utilizing administrative data by Kerola et al. [8], Tekin et al. [14], and Grellmann et al. [15]. A slight prevalence advantage for men over women has been noted in a retrospective study of the US population [30].

The European and global data presented are extremely difficult to compare due to methodological differences. The Polish data on PsA incidence in 2013 are between those presented in the northern and southern European papers. Despite the low incidence of PsA in 2021 found in our study is difficult to explain, we cannot exclude similar changes in epidemiology in other populations using similar study methods. In addition to methodological differences, the observed differences in incidence and prevalence may be influenced by differences in the prevalence of psoriasis in different populations. Psoriasis occurs in 0.47% of the population in Poland [16], much more frequently in Norway (3.8%−4.6%) [36], Germany (1.90%–2.51%) [37] and the USA (3%) [38], and much less frequently in Japan (0.34%) [39]. While our data do not directly assess treatment effectiveness or long-term outcomes, it is reasonable to speculate that improvements in PsA management may be contributing to the observed trends. Our data are also vital in terms of determining the level of access to innovative PsA treatment in Poland. In an earlier study, due to the lack of precise epidemiological data based on a constant number of PsA patients in the analysed timeframe, the estimated percentage of patients using biologic therapy/Janus kinase inhibitors in 2013, 2016, 2019 and 2022 was 1.4, 2.5, 4.5 and 8.7%, respectively [40]. Taking into account current epidemiological data and the number of patients treated in particular years, the percentage of patients treated innovatively should be recalculated to 2.5, 3.6, 6.2% in 2013, 2016, 2019, respectively, and as 9.2–12.1% in 2021–2022, which means better availability in this group of patients than previously estimated. Better availability may result in better patient prognosis at the population level. The increasing prevalence of PsA despite declining incidence likely reflects a complex interplay of factors, including not only potential improvements in treatment but also changes in diagnostic practices, increased awareness of the disease, and aging of the population. It is possible that better treatment options are contributing to increased survival rates among individuals with PsA. However, further research is needed to confirm this hypothesis and to quantify the precise impact of treatment on long-term outcomes and prevalence trends.

A strength of our analysis is that the results were compiled using data on all persons defined as PsA patients diagnosed and treated in the public health system in Poland between 2013 and 2021. In addition, a strict case definition, including two visits and prescription fulfilment, influenced the elimination of potentially unconfirmed diagnoses (treated by clinicians as suspected PsA but reported as PsA). To make the incidence study as robust as possible, we reviewed the incidence of specific ICD-10 codes three years before the analysis period and estimated the incidence in 2021 only after obtaining complete data for 2022. The importance of these results lies in the fact that, after excluding questionable cases and accounting for potential underestimation, we can confidently assert that the data presented are not overestimated.

Our study has certain limitations. The limited timeframe of the presented analysis may affect the interpretation of the trend. This analysis only includes patients treated by public medical services. It is possible that some patients did not receive public specialist services between 2012 and 2021 but only private medical care, which prevented their administrative identification as PsA patients. Notably, the proportion of PsA patients in Poland treated exclusively in private healthcare seems to be negligible. In contrast to other studies using administrative data, to avoid overestimation of the problem being studied, we decided to include the need for a relevant prescription in the patient definition. This definition may lead to the exclusion from the study of genuine PsA cases in several scenarios: it might have excluded patients in long-term remission either spontaneously or through successful treatment, who might not require ongoing pharmacologic therapy at a specific time and therefore would not meet our prescription criterion, even if they still carry the diagnosis, patients who experienced diagnostic delays or gaps in care, leading to periods where they don’t have both the required diagnostic codes and a current prescription and patients with psoriasis who were treated with disease-modifying therapies (conventional and targeted) and achieved early suppression of joint symptoms, preventing a subsequent diagnosis of PsA. However, it also excluded patients with joint complaints other than PsA. Restrictions in access to health care system services during the COVID-19 pandemic in 2020–2021, which occurred predominantly in low and middle-income countries (LAMI) [41], also to some extent in the rheumatology sector in Poland [40] may have negatively influenced the number of new PsA cases registered according to our methodology. While diagnostic and reporting delays likely contributed to this decline, our data do not allow for a detailed statistical adjustment or focused analysis of this specific period. This is a limitation of our study, and further research is needed to quantify the precise impact of the pandemic on PsA epidemiology in Poland. On the other hand, the inclusion of NSAIDs in the analysis, the primary treatment option for axial disease, may have resulted in an overestimation of the values because we could have classified patients with psoriasis and arthralgia as PsA, which the physician incorrectly identified as suspected PsA, although this seems of little consequence. While we have discussed potential contributing factors, including improved diagnostic accuracy and earlier biologic therapies, it is possible that the higher incidence reported in 2013 reflects an overestimation due to our methodology. At the beginning of the study period, the administrative database might have included prevalent cases (patients already diagnosed before 2013) alongside new diagnoses, potentially inflating the initial incidence rate. The application of our case definition (two visits with ICD-10 codes and at least one reimbursed prescription) might not fully differentiate between new and existing cases at the study’s commencement. The relatively stable incidence rates observed in the later years of the study are encouraging but must be viewed within the context of the limitations of the data source. Finally, interpreting studies using administrative data requires careful consideration as they may present limitations related to coding accuracy, changes in diagnostic practices, and the inherent challenges of differentiating between incident and prevalent cases, particularly at the beginning of the study period.

To our knowledge, the study presented is the first based on a very precise patient definition that includes separate visits and prescription fulfilment. Similar definitions have been used in recently published epidemiological studies on rheumatoid arthritis and juvenile idiopathic arthritis [42, 43]. Many factors may influence the epidemiology of PsA; however, the described differences in incidence and prevalence are unlikely to be explained solely by geographical location, genetic predisposition, or access to healthcare. The epidemiology of PsA appears to be changing and requires up-to-date, large-scale studies in different populations, preferably using similar methodological assumptions.

## Data Availability

The data in this study were obtained with the permission of the Ministry of Health of the Republic of Poland from electronic databases of the National Health Fund (NFZ). Datasets are not publicly available because they contain sensitive data at an individual level. Aggregated data may be requested from the Department of Analyses and Strategies in the Ministry of Health in accordance with the provisions on access to public information (a justification for the public interest is required).
